# The complete chloroplast genome sequence of *Liparis vivipara* (Orchidaceae)

**DOI:** 10.1080/23802359.2019.1624638

**Published:** 2019-07-11

**Authors:** Diyang Zhang, Ding-Kun Liu, Yang Hao, Si-Ren Lan, Zhong-Jian Liu

**Affiliations:** a Key Laboratory of National Forestry and Grassland Administration for Orchid Conservation and Utilization at College of Landscape Architecture, Fujian Agriculture and Forestry University, Fuzhou, China;; b Fujian Colleges and Universities Engineering Research Institute of Conservation and Utilization of Natural Bioresources, College of Forestry, Fujian Agriculture and Forestry University, Fuzhou, China

**Keywords:** Chloroplast genome, phylogenetic, Illumina sequencing, *Liparis vivipara*

## Abstract

*Liparis vivipara* is a terrestrial orchid distributes in southwestern China. In this study, we reported the first complete chloroplast genome of *L. vivipara*. The whole genome was 158,329 bp, consisting of a pair of inverted repeats (IR 27,043 bp), a large single-copy region (LSC 85,950 bp), and a small single-copy region (SSC 18,293 bp). The complete genome contained 132 genes, including 77 protein-coding genes, 38 tRNA, and 8 rRNA genes. The overall GC content of the whole genome was 36.9%. A maximum-likelihood phylogenetic analysis demonstrated a close relationship between *L. vivipara* and *L. loeselii*.

The genus *Liparis* (Orchidaceae) are terrestrial, epiphytic or rarely lithophytic, comprising approximately 400 species worldwide with over 50 species distributed in China (Li and Yan 2013; Huang et al. 2018; Liang et al. 2019). *Liparis vivipara* is a terrestrial species distributes in evergreen, broad-leaved forest at elevation of 1350 m, Yunnan Province, southwestern China (Huang et al. 2018). Fieldwork by Huang et al. (2018) showed that *Liparis vivipara* has only four known populations, each includes less than 30 individuals, which indicates a critically endangered status base on World Conservation Union Red List Categories and Criteria (IUCN 2012). It also has a unique feature that its upper older pseudobulbs often bearing black bulbils (Huang et al. 2018). In light of these findings, a comparative analysis regarding its phylogeny is requiring to further investigate its evolution history and population genetic studies. Therefore, we reported a complete chloroplast genome of *Liparis vivipara* to better understand the generic delimitations between *Liparis* and related genera as well as contribute to an effective conservation strategy for *L. vivipara.*

The total genomic DNA was extracted from fresh leaves using a modified CTAB method (Doyle and Doyle 1987) and sequencing was carried out by the Illumina pair-end technology. The leaf sample was collected from Malipo County, Yunan Province, China (23°7′11.9″N, 104°42′E). The DNA was stored at Fujian Agriculture and Forestry University (Voucher specimen: LP-01, FAFU). The clean reads were aligned to *Dendrobium officinale* (GenBank accession No. LC348521) (Zhu et al. 2018) and assembled by GetOrganelle pipe-line (Jin et al. 2018). The assembled chloroplast genome was annotated using DOGMA (Wyman et al. 2004), and corrected by Geneious 11.1.15 (Kearse et al. 2012). The physical map of the new chloroplast genome was generated using OGDRAW (Lohse et al. 2013). The annotated complete chloroplast genome was submitted to GenBank with accession number MK862100. The complete chloroplast genome of *L. vivipara* is 158,329 bp in length, comprising a large single-copy (LSC) region of 85,950 bp, a small single-copy (SSC) region of 18,293 bp, and two inverted repeat (IR) regions of 27,043 bp. The new sequence contained 132 genes in total, including 77 protein-coding genes, 8 rRNA genes, and 38 tRNA genes. The base composition of *L. vivipara* cp genome was uneven (31.1% A, 18.8% C, 18.2% G, 32% T) with an overall GC content of 36.9% and the corresponding values of the LSC, SSC, and IR regions reaching 34.6, 29.6, and 43.1%, respectively.

To investigate the phylogenetic position of *L. vivipara*, 17 complete cp genome of species from Epidendroideae and 2 species of Orchidoideae were aligned using HomBlocks pipeline (Bi et al. 2018). RAxML-HPC Black-Box version 8.1.24 (Stamatakis et al. 2008) was used to construct a maximum likelihood tree with *Oncidium hybrid cultivar* and *Cymbidium aloifolium* as outgroup. The branch support was computed with 1000 bootstrap replicates. The ML tree analysis indicated that *L. vivipara* and *L. loeselii* cluster together with 100% bootstrap support (Figure 1).

**Figure 1.**
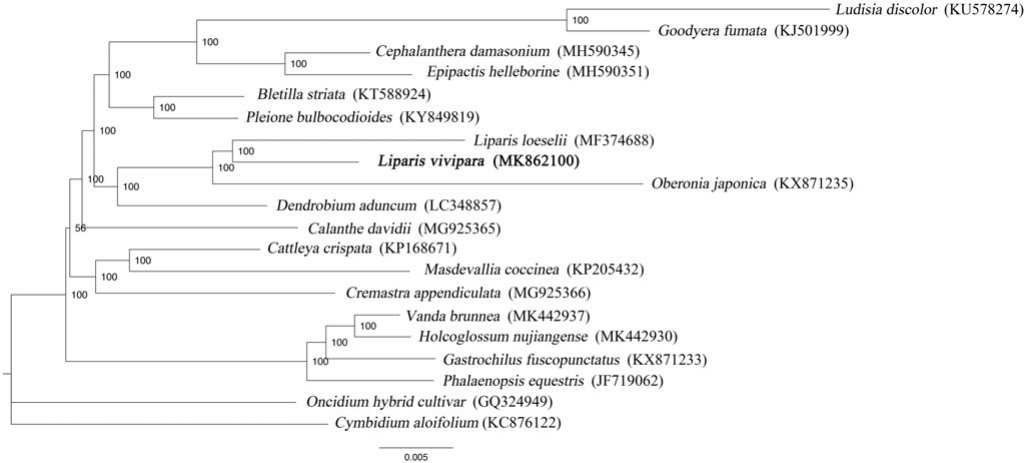
Maximum likelihood tree based on the complete cp genome sequences of 20 species from the Orchidaceae, with *Oncidium hybrid cultivar* and *Cymbidium aloifolium* as outgroup. The bootstrap value is shown on each node and the position of *Liparis vivipara* is in bold.
